# A modeling framework for the establishment and spread of invasive species in heterogeneous environments

**DOI:** 10.1002/ece3.2915

**Published:** 2017-09-08

**Authors:** Audrey Lustig, Susan P. Worner, Joel P. W. Pitt, Crile Doscher, Daniel B. Stouffer, Senait D. Senay

**Affiliations:** ^1^ Bio‐Protection Research Centre Lincoln University Lincoln New Zealand; ^2^ 8i Wellington New Zealand; ^3^ Lincoln University Lincoln New Zealand; ^4^ University of Canterbury Christchurch New Zealand; ^5^ INSTEPP University of Minnesota Minneapolis MN USA

**Keywords:** heterogeneous landscape, invasive species, landscape metrics, landscape patterns, population dynamics, spatial simulations, stratified dispersal

## Abstract

Natural and human‐induced events are continuously altering the structure of our landscapes and as a result impacting the spatial relationships between individual landscape elements and the species living in the area. Yet, only recently has the influence of the surrounding landscape on invasive species spread started to be considered. The scientific community increasingly recognizes the need for broader modeling framework that focuses on cross‐study comparisons at different spatiotemporal scales. Using two illustrative examples, we introduce a general modeling framework that allows for a systematic investigation of the effect of habitat change on invasive species establishment and spread. The essential parts of the framework are (i) a mechanistic spatially explicit model (a modular dispersal framework—MDIG) that allows population dynamics and dispersal to be modeled in a geographical information system (GIS), (ii) a landscape generator that allows replicated landscape patterns with partially controllable spatial properties to be generated, and (iii) landscape metrics that depict the essential aspects of landscape with which dispersal and demographic processes interact. The modeling framework provides functionality for a wide variety of applications ranging from predictions of the spatiotemporal spread of real species and comparison of potential management strategies, to theoretical investigation of the effect of habitat change on population dynamics. Such a framework allows to quantify how small‐grain landscape characteristics, such as habitat size and habitat connectivity, interact with life‐history traits to determine the dynamics of invasive species spread in fragmented landscape. As such, it will give deeper insights into species traits and landscape features that lead to establishment and spread success and may be key to preventing new incursions and the development of efficient monitoring, surveillance, control or eradication programs.

## 1 Introduction

Preventing the spread of invasive species into new habitats requires an awareness of the types of species that might pose a threat to an ecosystem, and which ecosystems are especially vulnerable to invasion in the face of increasing land‐use and climate change. Theory indicates that the distribution and spread of invasive species is the result of a complex combination of factors (Catford, Vesk, White, & Wintle, 2011). These factors include the size and frequency of introduction (propagule pressure), species‐specific traits that are thought to confer high fitness such as high reproductive and efficient dispersal abilities, and the abiotic and biotic characteristics of the recipient ecosystems that may limit or facilitate the establishment of invasive species. Surprisingly, dynamic risk predictions of establishment and spread typically lack explicit considerations of the interaction between these multiple drivers of invasion (Catford et al., 2011; Gallien et al., 2014). In particular, spatiotemporal predictions of establishment and spread, across different species demography and dispersal characteristics and environmental conditions, are very few (Dormann et al., 2012; Franklin, 2010; Guisan & Thuiller, 2005; Huntley et al., 2010; Schurr et al., 2012; Thuiller et al., 2013; Worner, 1994).

Early progress in the development of models of establishment and spread was divided into approaches used for modeling large‐scale species distribution versus those for modeling local‐scale population spread (Hastings et al., 2005; Hui, Krug, & Richardson, 2011). Broad‐scale projections of species’ distribution, which have dominated the recent literature, are largely based on static approaches linking current species occurrences to environmental variables such as temperature, precipitation, and resource distribution (Guisan & Zimmermann, 2000). These models can be used to project distribution and impacts from future land use or climate scenarios (Thomas et al., 2004), yet typically overlook important demographic and dispersal processes. On the contrary, mechanistic models of spread, such as reaction–diffusion processes (Fisher, 1937; Skellam, 1951), integro‐difference equations (Kot, Lewis, & van den Driessche, 1996), matrix models (Caswell, 2001; Ramula, Knight, Burns, & Buckley, 2008), or metapopulation models (Hanski, 1999; Hanski & Ovaskainen, 2000), incorporate, to differing degrees, both demography and dispersal processes as their basis, linking the spatial location of reproducing individuals to the spatial location of their parents through the definition of a dispersal kernel. However, at present, it is not clear how an analytically tractable model can formally represent a real landscape in the form of a quality function, and thus, consideration of biological and geographical realism is still limited. The general evolution of combining demographic, dispersal, and spatial complexity in spatial population models has resulted in mechanistic model of spread integrated within geographical information systems (GIS) and are often referred as process‐based species distribution modeling (SDM; Nehrbass & Winkler, 2007; Pitt, Worner, & Suarez, 2009; Renton, Savage, & Chopard, 2011; Guichard, Kriticos, Leriche, Kean, & Worner, 2012; Bocedi et al., 2014; Merow, Latimer, et al., 2014; Lurgi, Brook, Saltré, & Fordham, 2015). These models are thought to be more robust to extrapolation to novel habitat and climate conditions because they rely on the characterization of processes regulating the probability of a population surviving to reproduce and disperse in response to local environment conditions. Therefore, such models account for the effect of landscape characteristics on the mobility and survival of invading species (Ewers & Didham, 2006; Pitt et al., 2009).

Despite much progress, understanding how life‐history traits and landscape characteristics, such as the amount and spatial distribution of habitat cover classes, interact to determine the dynamics of invasive species spread is currently confined to broad generalization (Catford et al., 2011; McConkey et al., 2012). Particular issue is that most empirical and theoretical studies evaluate questions and hypotheses about the role of landscape structure within a single landscape and thus provide no replication (but see With, 2002; Vilà & Ibáñez, 2011; González‐Moreno, Pino, Gassó, & Vilá, 2013). Additionally, the majority of these studies focus on a single species and thus do not provide insight into trait variability in the same landscape (but see Catford et al., 2011; Robinet et al., 2012; Wang & Jackson, 2014). More fundamentally, in a thorough review of contemporary plant dispersal ecology, Robledo‐Arnuncio, Klein, Muller‐Landau, and Santamaría (2014) emphasized that spatially explicit spread modeling is confronted with difficulties arising from a lack of a conceptual framework to investigate the relationships between quantitative measures of spatial heterogeneity and the spread of species.

The primary aim of this study was therefore to present a general modeling framework that allows for a systematic investigation of the impact of landscape structure on invasive species establishment and spread. The essential parts of the framework are (i) an individual‐based spatially explicit model (MDIG) that allows population dynamics and dispersal to be modeled in GIS (Pitt, 2008), (ii) a landscape generator that allow replicated landscape patterns with partially controllable spatial properties to be generated, and (iii) landscape metrics that depict the essential aspects of landscape with which dispersal and demographic processes interact. Such a framework will give deeper insights into species traits and landscape features that lead to establishment and spread success and may be key to preventing new incursions and the development of efficient monitoring, surveillance, control and eradication programs. The framework is unique in two key aspects. First, it includes the capability for much greater realism when modeling reproduction and dispersal processes as it accounts for that interindividual variability and key stochasticities in demographic and dispersal processes. Second, the framework explicitly relates demographic and dispersal processes to the landscape in which these processes occur, using either the open‐source GIS program GRASS (http://grass.osgeo.org) or computer‐generated landscapes. The extended framework therefore offers possibilities for a broad range of simulation‐based modeling experiments, from basic theoretical investigations of invasion dynamics in fragmented landscapes, to strategic modeling of spatiotemporal species distribution and management options.

## 2 Model Description

### 2.1 Component 1: A spatially explicit dispersal model

Recent advances in establishment and dispersal modeling have integrated population dynamic processes into SDMs, to simulate the response of an individual or a population to environmental conditions to predict abundance, cover, or probability of presence of a species at a given location (Lurgi et al., 2015). Among these models often referred as process‐based SDMs, the modular dispersal model (MDiG) is a spatially explicit, stochastic spread model originally developed as a freely available, open‐sourced application by (Pitt, 2008, http://github.com/ferrouswheel/mdig). MDiG uses GRASS‐GIS raster maps to represent either the presence/absence or abundances of the species under study in raster cells. Initial distribution data can be imported or defined by the user. The model architecture was designed to be extensible to cope with many different taxa, characterized by different population dynamics and dispersal strategies, over realistic landscapes. At a population scale, a demographic submodel provides different levels of complexity to mimic the fate of individual organisms, by simulating the life‐history events of birth and death. At the landscape scale, a dispersal submodel provides explicit rules that determine the pattern of dispersal for each individual. The model definition file is specified in a file formatted in XML that defines when and how demography, dispersal, simulation results, and other model aspects are specified. The flexibility of the model facilitates future research by allowing influences on growth and dispersal, additional to those investigated in this study and previous work, to be easily incorporated and investigated (Lurgi et al., 2015; Pitt, 2008). All these features will encourage researchers to use a single (or a small set of) modeling platform(s) rather than having a large set of user‐ or group‐specific tools, thus limiting the duplication of effort in software development, reducing model‐based errors through improvement (Lurgi et al., 2015).

#### 2.1.1 The population submodel

The growth module, “r.mdig.growth”, is designed to represent local growth or the number of individuals within each raster cell, from one time step to another. The definition of a time step is up to the user, but applies to the entire model. The carrying capacity parameter indicates the maximal number of individuals in a cell. It can be specified as a global value, for each land‐cover category or for each cell of the map, to accommodate for spatiotemporal variation in habitat quality. The population growth dynamic is determined by a difference equation chosen and parameterized by the user. The options include the logistic growth (Verhulst, 1838), Beverton–Holt equation (Beverton & Holt, 1957), Ricker equation (Ricker, 1958), Neubert equation (Neubert, 1997), Wang equation (Wang, Kot, & Neubert, 2002), Keitt equation (Keitt, Lewis, & Holt, 2001), or the user can add more functions as the need arises. Population‐based simulations with different life stages can apply a matrix‐based population model using the life‐stage module, “r.mdig.agepop”, for which fecundity, survival, development, and dispersal can be stage/age dependent. The module is designed to account for growth and dispersal age dependency.

#### 2.1.2 The dispersal submodel

##### Local dispersal

The neighborhood module, “r.mdig.neighbour”, is designed to represent local spread or a diffusion process based on random walks to surrounding adjacent cells. The proportion of individuals that spread from any cell can be specified as a parameter. Both the shape, which defines the direction of the neighborhood of a cell (east, north, west, south), and the range, which predetermines the extent of the neighborhood (2 or 4 cells), are used to represent local random movement of individuals.

##### Long‐distance dispersal events

The kernel module, “r.mdig.kernel”, is designed to represent long‐distance dispersal events, resulting, for example, from wind disturbances, animal dispersal, or through human transportation. A Poisson process is used to approximate the number of long‐distance dispersal events that are generated from an occupied cell. The user can parameterize a Cauchy (Shaw, 1995) or exponential (Mollison, 1972) dispersal kernel to determine the distances travelled from the occupied site (Levin, Muller‐Landau, Nathan, & Chave, 2003; Nathan et al., 2002). Finally, a uniform distribution in the range of [0, 2π] is sampled to determine the direction of each generated long‐distance event (Pitt, 2008). The relative contribution of multiple vectors to particular dispersal pathways can be investigated using dispersal kernels characterized by mixed probability distributions (Gilbert, Grégoire, Freise, & Heitland, 2004).

#### 2.1.3 Postdispersal survival

The survival module, “r.mdig.survival”, allows the species–landscape interactions to be incorporated. The user specifies a habitat suitability map that can be either realistic maps based on known habitat suitability generated in GIS (Pitt, 2008) or artificial maps produced by a landscape generator, in the form of survival probability maps ranging from 0 to 1, reflecting the difficulty that populations have in establishing within each raster cell. The framework is asynchronous. The individuals modeled through the local dispersal, and dispersal kernel modules are passed through the survival module to determine the population in each cell surviving to the next simulation step based on the underlying suitability value. It is also possible to provide a single survival value if the landscape is homogeneous, such as with a monoculture in an agricultural field or glasshouse (Pitt, 2008).

### 2.2 Component 2: Modeling habitat suitability

#### 2.2.1 Generating habitat suitability maps or the survival layer

There are numerous ways to create habitat suitability maps, and they can be based on a wide range of data (Guisan & Thuiller, 2005; Guisan & Zimmermann, 2000). The most common methods are based on static approaches linking current species distribution to environmental variables such as climate, vegetation, or human disturbance (Guisan & Thuiller, 2005; Thuiller et al., 2008). Others used phenological models (Pitt, Régnière, & Worner, 2007; Régnière & Nealis, 2002) or expert opinion (Harris, 2002). In a thorough review of the ecological principles and assumptions underpinning habitat suitability modeling, Guisan and Thuiller (2005), Araújo and Guisan (2006), and Elith, Leathwick, and Hastie (2009) have all highlighted the key steps in good habitat–suitability–modeling practice including gathering the relevant data, dealing with correlated predictor variables, selecting an appropriate modeling algorithm, fitting and evaluating the model performance and predictive performance.

#### 2.2.2 Computationally generated landscapes

Another option for creating habitat suitability maps comprises using a landscape simulator that provides a framework for generating replicated landscape patterns with partially controllable spatial properties, particularly with respect to their composition and configuration of components (Turner, 1990; With & King, 1997). When combined with a population dynamic model such as MDiG, these artificial landscapes serve as a template to systematically investigate the effect of landscape structure in fragmented and heterogeneous landscapes (Turner, 2005). The successful application of computer‐generated landscapes has also led to the development of software designed to create them using a variety of algorithms. Examples include QRULE (Gardner & Urban, 2007), SIMMAP (Saura, 2003), as well as software packages such as the *ecomodtools* package for R (Chipperfield, Dytham, & Hovestadt, 2011) or the python‐based NLMpy package (Etherington, Holland, & O'Sullivan, 2014). These tools provide easy integration with geospatial data and can be integrated within MDiG, allowing the design of models ranging from a very simple static spatial establishment and spread model to very complex dynamic ones.

### 2.3 Component 3: Characterization of landscapestructure

The ability to quantitatively describe landscape structure is a prerequisite to detect changes and to investigate the relationship between landscape structure, and demographic and dispersal processes. The plethora of metrics available means that an exhaustive review of all published metrics is beyond the scope of this study. To date, the most comprehensive overview of formulae and domains of traditional metrics has been provided by McGarigal, Cushman, and Ene (2012). The general perception is that there are three important problems associated with the use of landscape metrics. They are (i) a high degree of correlation in between the metrics themselves, (ii) ambiguous responses to different spatial processes, and (iii) sensitivity to changes in spatial scale. Quantifying the specific effect of habitat configuration on spread success, for example, is difficult because many configuration metrics are correlated with the percentage of habitat in the landscape (Kupfer, 2012). Such limitations can often be addressed, or put in perspective, through careful data manipulation, analysis, and interpretation (Kupfer, 2012; Lustig, Stouffer, Roigé, & Worner, 2015; McGarigal et al., 2012; Uuemaa, Mander, & Marja, 2013). To calculate landscape metrics, computer programs have been developed such as Fragstats (McGarigal et al., 2012), as well as the python‐based vLATE and Patch Analyst 4.1 modules implemented on ArcGIS (Rempel, Kaukinen, & Carr, 2012), or the plugin LecoS (http://www.qgis.org/en/site/) for the Quantum GIS freeware and the two open‐source modules r.le and r.li (Rocchini et al., 2013) implemented in GRASS‐GIS.

## 3 Example of Applications

### 3.1 The effect of spatial heterogeneity on the establishment and spread of *Pieris brassicae*

We use the invasive great white butterfly, *P. brassicae*, to investigates how different representation of realistic spatial patterns can change the final projected distribution of the spread of this species in Nelson area, New Zealand. The chosen spatial extent roughly measures 35 × 40 km around Nelson port, which is suspected to be the introduction site of *P. brassicae*. The cell resolution was set to 50 m and covers a heterogeneous area in terms of both land cover and climatic composition (Section 1.2 in Supporting Information).

**Figure 1:**
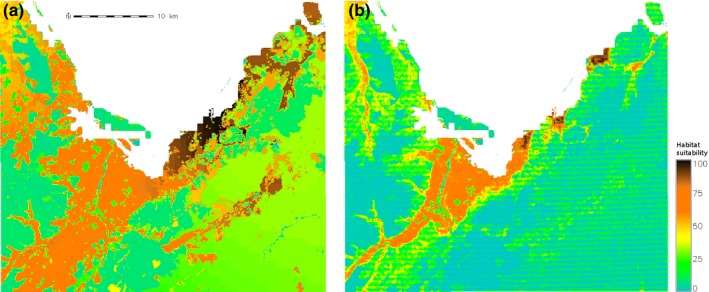
Two suitability maps used to build the survival layers of *Pieris brassicae*. (a) Survival layer, LS_1_, including the necessary base temperature to complete the *P. brassicae* life cycle within a season (GDD) and the New Zealand Land Cover Dataset LCDB2 (LCC), (b) Survival layer, LS_2_, comprised components of the first landscape, as well as a raster layer that captures local elevation variation (LEV)

In this case study, we focus on a dynamic presence/absence model. The average frequency of long‐dispersal events emerging from each occupied cell was drawn from a Poisson process (λ = 11,894 m), while the median distance travelled was approximated by a Cauchy distribution (*x*_0_ = 24,752; *f* = 0.41; Senay, 2014). Two suitability maps, given as 0–1 survival probability, were developed to investigate the effect of landscape composition and configuration on invasive species spread (Figure 1—Section 1.3 in Supporting Information). The first landscape (LS_1_) included a raster layer that reflects the necessary base temperature to complete the *P. brassicae* life cycle within a season (Senay, 2014), and the New Zealand Land Cover Dataset LCDB2 (Ministry for the Environment, NZ, 2004) that allowed the land covers that are suitable to *P. brassicae* to be differentiated. The second survival layer (LS_2_) comprised components of the first landscape, as well as a raster layer that captures local elevation variation. The rationale for incorporating local elevation gradients is that migrating *P. brassicae* have been seen flying following altitudinal ridges (personal communication, Department of Conservation, NZ). Both survival layers were characterized by three commonly used landscape metrics: a measure of the proportional abundance of habitat in the landscape (PLAND), a simple measure of fragmentation (NP), and a measure of the degree of connectedness among habitat patch on a landscape (CONNECT; McGarigal et al., 2012). These metrics were calculated for the landscape as a whole, and, respectively, for the suitable areas (survival probability > .5), less suitable areas (survival probability ∈[.1,.5[), and marginal areas (survival probability < .1; Section 1.3.4 in Supporting Information).

**Figure 2:**
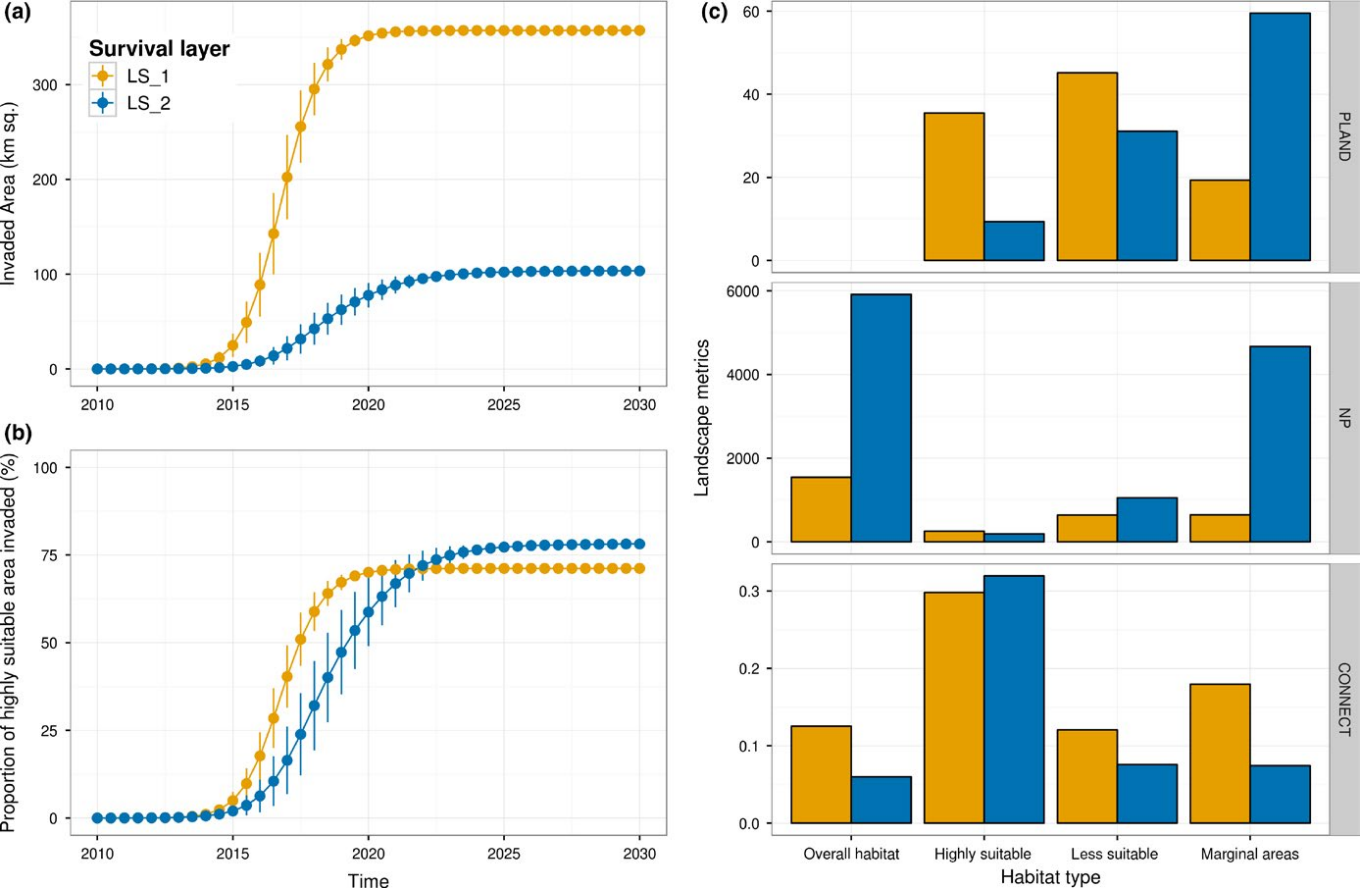
*Pieris brassicae*, range expansion over the two suitable layer LS_1_ (yellow) and LS_2_ (blue). Subfigures (a) and (b), respectively, represent the total surface area invaded and the proportion of highly suitable patches occupied as a function of time. The mean value and standard deviation of the 500 simulation replicates are represented. Subfigure (c) represents a measure of the proportional abundance of habitat type in the landscape (PLAND), the number of patch for each class type (NP), and a measure of the degree of connectedness among class type on a landscape (CONNECT). These metrics were calculated for the landscape as a whole, and, respectively, for the highly suitable areas (survival probability > .5), less suitable areas (survival probability ∈[.1,.5]), and marginal areas (survival probability < .1). When calculated for the landscape as a whole, the three metrics measure the aggregate properties of every habitat patch in the landscape, When calculated for a specific habitat type (e.g., highly suitable habitat type), the three metrics measure the aggregate properties of the habitat patches belonging to this particular habitat type (e.g., CONNECT for highly suitable habitat measure the average connectivity between highly suitable patches)

For both survival layers, the area occupied remains relatively low during the first seven years of simulation before the surface invaded increased exponentially toward saturation of the considered spatial extent (Figure 2a). Recording the elevation gradients (LS_2_) reduced by a factor of three, the proportion of highly suitable sites (PLAND) in the landscape (Figure 2c), and resulted in an apparent decrease in the surface invaded (Figure 2a). Interestingly, the survival layer that included elevation gradients showed, on average, a higher level of fragmentation (NP) and a lower degree of connectedness among habitat type (CONNECT). However, recording elevation had a relatively low effect on the spatial arrangement of suitable patches that remain highly connected (CONNECT) compared with less suitable area (Figure 2c). The reclassification scheme used in LS_2_ did not limit access to suitable habitat patches, and in both scenarios, the species was able to invade about 75% of the suitable areas in the landscape over the simulation time frame (Figure 2b). The results show the importance of three different landscape metrics for robust habitat characterization. The metrics may be considered as “landscape signature” as they serve as discriminators of land cover/land uses within the study area.

### 3.2 Impact of landscape structure on mean population size and mean dispersal distances

**Figure 3:**
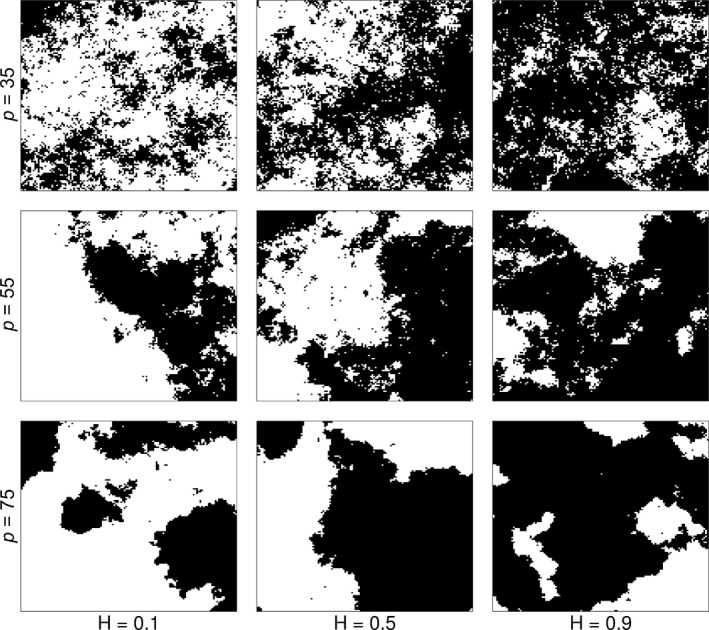
Example of survival layers used in *Lymantria dispar* dispersal model. The landscapes were simulated across a three‐step gradient of habitat fragmentation (*H*) and a three‐step gradient of habitat amount (*P*). These landscapes are used to test the effect of landscape structure on the establishment and spread of an invasive species

We investigated how change in habitat composition and configuration can affect population density and the dispersal abilities of the Asian gypsy moth, *Lymantria dispar*. We used the computer program Qrule 4.2 to generate binary (suitable, unsuitable) landscapes, in which habitat configuration (spatial autocorrelation, *H* = .3, .5, .7) and habitat amount (*P* = 35%, 55%, 75%) can be systematically and independently controlled (Gardner & Urban, 2007; Figure 3—Section 2.2 in Supporting Information). The cell resolution was set to 10 km to approximate the median distance of local movements of *L. dispar* as shown in Johnson, Liebhold, Tobin, and Bjørnstad (2006) and Liebhold, Halverson, and Elmes (1992) (Section 2.3 in Supporting Information). The average frequency of long‐dispersal events emerging from each occupied cell was drawn from a Poisson process (λ = 3 or 5 cells), while the median distance travelled was approximated by a Cauchy distribution (*x*_0_ = 1; *f* = 0.05; Jankovic & Petrovskii, 2013; Section 2.4 in Supporting Information). The initial dispersal site was arbitrarily set in the landscape. Following Johnson et al. (2006) and Liebhold and Bascompte (2003), the local density of *L. dispar* was approximated by a deterministic Allee logistic growth model (Section 2.5 in Supporting Information).

Each landscape was characterized by two commonly used landscape metrics: a measure of the proportional abundance of each class type in the landscape (PLAND) and a measure of the degree of connectedness among class type on a landscape (CONNECT; Section 2.2 in Supporting Information). Finally, MDiG was used to quantify the dependency of population density (*d*) and rate of spread (ROS) on the landscape characteristics (PLAND and CONNECT), the population traits dispersal ability (dist) and intrinsic rate of increase (*r*).

**Figure 4:**
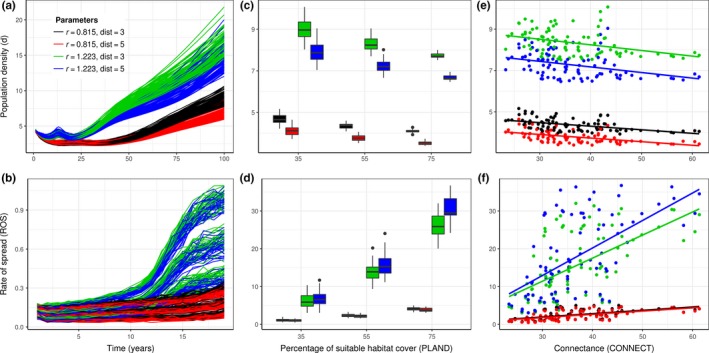
European gypsy moth, *Lymantria dispar*, range expansion. The first column shows (a) the population density and (b) the rate of spread (no. of new occupied cells/year) over a period of 100 years. Each time series depicts one of the 500 simulation replicates for a different combination of intrinsic rate of increase (*r*) and long‐distance dispersal ability (dist) of the species. In the middle, (c) the average population density (*d*) and (d) rate of spread (ROS) for each combination of *r* and dist are represented as a function of the percentage of suitable habitat in the landscapes (PLAND), while the column on the right (e,f) represents the same output as a function of the connectivity index (CONNECT)

For a single introduction of five individuals into the landscape, the simulated average population density remains relatively low during the first 25 years before the population density increases exponentially towards the habitat carrying capacity (*K* = 100; Figure 4). The rate of spread is characterized by an initial phase with a relatively low spread. New sites are further colonized only after the population locally grows in the newly invaded sites (*t* = 25) and starts to produce new propagules that can sustain the wave of advancement (Figure 4). The existence of such dynamics in rate of spread has long been reported and may occur for several reasons (Hastings et al., 2005). For example, individuals must overcome Allee effects that may constrain growth in newly invaded sites before generating propagules for further invasion, potentially imposing limits on totally unregulated spread (Smith et al., 2014).

Increasing the percentage of suitable habitat cover in the landscape (PLAND) and the degree of connectedness among suitable patches on a landscape (CONNECT) resulted, on average, in a more prolific spread but reduced the local density of the population (Figure 4). This asymmetry in the response to changes in the structure of the landscape suggests that species that have limited dispersal opportunities tend to maximize their populations locally but will be limited for establishing a population over a large area. On the other hand, species that have high dispersal opportunities may spread but face the added risk of not establishing or going extinct due to lower population density and consequent Allee effects. Interestingly, the response of the species to changes in habitat structure was independent of the intrinsic dispersal abilities of the species. However, species with a higher intrinsic rate of increase (*r*) systematically outperformed species with a lower rate of intrinsic increase.

## 4 Discussion and Conclusion

The conceptual framework introduced in this study relies on a combination of methods, namely the use of a spatially explicit spread simulator, a landscape generator, and landscape metrics. In this study, we have shown these elements can be combined in an iterative process, to provide quantitative information about the relationship between demography and dispersal processes, and the environment in which they occur.

Demography and dispersal processes are clearly key determinants of species’ spatial dynamics and responses to rapid environmental change. However, insufficient representation of dispersal at the landscape scale is still a major limitation in many approaches used for SDMs (Baguette & Van Dyck, 2007; Clobert, Baguette, Benton, Bullock, & Ducatez, 2012; Travis et al., 2013). In contrast, MDiG allows demography and dispersal to be modeled explicitly to explore responses to landscape structure (Pitt, 2008). The model is very flexible in that it can be applied at multiple spatiotemporal scales and can be easily modified for species with structurally different demographic and dispersal behaviors thus generalizing its use to many different taxa. It also allows the manipulation of natural and/or anthropogenic landscapes to test predictions regarding landscape modification or regional climate change for invasive species management and conservation purposes. In particular, process‐based models such as MdiG differ from correlative models in that they consider how the environment constrains physiological performance at a given location (Evans, Diamond, & Kelly, 2015; Pacifici et al., 2015). MdiG contains explicitly defined parameters that have clear ecological interpretation (Pitt, 2008). As such, MDiG can facilitate mechanistic explanations of the factors underlying responses to environmental change, while clarifying the roles of important environmental and landscape influences. The spread simulator was integrated and extended to allow change in the structure of the landscape to be described and quantified by means of landscape metrics. These metrics provide a unique means of investigating how small‐grain landscape characteristics, such as habitat size and habitat connectivity, interact with life‐history traits to determine the dynamics of invasive species spread in fragmented landscapes.

MDiG was initially developed to support strategic forecasts of spatiotemporal invasive species distribution and management options (Pitt, 2008). Accurately estimating the distribution of an invading organism at any time in the future, including the time it takes to reach an equilibrium within its new environment, is of paramount importance for planning eradication strategies or even to decide whether any eradication effort is necessary or possible (Venette, 2015). When used for spread forecast purposes, MDIG integrates specific characteristics of the studied species, for example, specific dispersal strategies or habitat requirements, to obtain “realism”. A particular challenge for establishment and dispersal modeling is to decide the appropriate extent, resolution, and level of landscape details over which dispersal studies are carried out (Venette, 2015). A good example from this research was that recording the elevation gradients for investigating the spread of *P. brassicae* in New Zealand reduced by a factor of three the proportion of highly suitable sites in the landscape and resulted in an apparent decrease of the surface invaded. The most important implication of such a result is that any overestimation of future dispersal potential, by incorrectly identifying the landscape factors that might constrain species establishment and spread, might incorrectly discourage an eradication attempt (Epanchin‐Niell, Haight, Berec, Kean, & Liebhold, 2012; Senay, 2014; Venette, 2015). In particular, overestimation of establishment and spread rates increases the likelihood that an area will be covered by biosecurity measures (Epanchin‐Niell, Brockerhoff, Kean, & Turner, 2014; Hauser & McCarthy, 2009; Holden, Nyrop, & Ellner, 2016). If policymakers assume the possibility of harm is certain due to high establishment and spread rates estimates, and they act to mitigate the invasion, they could be wrong and have committed suboptimal allocation of resources and investment. Similarly, if policymakers fail to act because establishment or spread rates have been underestimated—and they are wrong—then the failure to act could be catastrophic. In this study, we have shown that selective recoding of certain areas of the landscape based on species attributes can help optimize the level of landscape details required for reliable projections of species spread case by case. Nevertheless, the result of the simulations remain closely linked to the choice of the parameters rather than providing any insight into general principles.

In this research, however, a more holistic approach was adopted where the patterns of invasion generated by multiple species scenarios (variation in intrinsic growth rates and dispersal abilities) within different landscape structure were used to infer key drivers of population density and spread, using *L. dispar*, as a case study. These relationships between life‐history traits and landscape characteristics were evaluated for their generality and robustness via the manipulation of computationally generated landscapes with known landscape structure. A key aspect of this assessment was to identify which model parameters were likely to have a large impact on population density and spread estimates. The analyses in this study were built around a series of relatively simple assumptions regarding the characteristics of the species and the environment, such as a random walk for approximating local diffusion of the insects, a single Cauchy distribution for approximating long‐distance dispersal events or a binary distinction of suitable and unsuitable habitat. Despite this simplicity, the model as a whole is structurally complex and produced a variety of plausible range expansion dynamics that remain to be tested empirically. For example, the response of the species to changes in habitat structure was independent of the intrinsic dispersal abilities of the species. However, species with a higher intrinsic rate of increase systematically outperformed species with a lower rate of intrinsic increase for all landscapes considered. This result suggests that spread rate is more strongly related to intrinsic rate of increase, which determines the total number of individuals participating in dispersal, than it is to a species’ intrinsic dispersal ability. This suggestion also supports the study by Cassey, Prowse, and Blackburn (2014) which also identified demographic traits to be the most important factor influencing the probability of invasion of exotic birds. From an invasive species management perspective, this result suggests that priority should be placed on species with high intrinsic rate of increase and that eradication programs should focus on limiting reproductive stages as a priority. Ultimately, a generalized and exhaustive study that could elucidate a possible relationship between species attributes and mode of dispersal with optimum landscape resolution, configuration, and composition is greatly needed.

What is encouraging for future studies is that the availability of movement data is increasing rapidly, particularly with data of long‐distance dispersal in heterogeneous landscapes as well as with meaningful characterization of average growth and dispersal patterns across temporal scales (Cagnacci, Boitani, Powell, & Boyce, 2010; Morales et al., 2010; Robledo‐Arnuncio et al., 2014). High‐quality data of multiple species growth and movement across complex landscapes will allow better parameterization of the framework and better representation of population growth, density, rate of spread and trajectories of invasive species over different taxonomic groups and spatial scales, in environmentally and demographic explicit contexts. In turn, the framework can help to generate hypotheses to be tested empirically and determine how these hypotheses scale over time and space. A set of research questions for which a modeling framework such as MDiG could be used to help progress our understanding of the establishment and spread of invasive species in heterogeneous landscapes has been proposed in Lustig (2016). They include questions related to the relative contribution of multiple spread vectors to rate of spread, the implication of landscape‐dependent variation on demography and dispersal ability or the implications of demographic and environmental stochasticity on rate of spread. Choosing the appropriate degree of abstraction of species demography, dispersal, propagule pressure, and ecosystem characteristics to keep a balance between maintaining reality and reducing model complexity, is a fundamental challenge to establishment and spread modeling (Lurgi et al., 2015; Merow, Smith, et al., 2014; Venette, 2015). Thuiller et al. (2008) suggested that such a decision is scale dependent. Complex models are likely to be more accurate at finer resolutions, whereas simple models are likely to offer useful and parsimonious solutions at broader scales. Yet, the development of complex models are necessary not only to help understand the relative importance of different drivers and their interactions on the population density and spread of invasive species, but also as an aid to optimize the trade‐off between precision and model complexity.

## Conflict of Interest

None declared.

## Data Accessibility

MDIG software and manuals can be found in the Github repository https://github.com/ferrouswheel/mdig. The data used in this study can be found in the Github repository https://github.com/AudreyL/mdig-casestudy
