# Supplementary material for: A modeling framework for the establishment and spread of invasive species in heterogeneous environments
Source: Ecol Evol. 2017 Sep 8;7(20):8338–48. doi: 10.1002/ece3.2915 (PMC5648669; doi:10.1002/ece3.2915)
Supplement: Supplementary file 1 [file ECE3-7-8338-s001.pdf]

Supplemental Materials

1 Modelling dynamic range expansion of *Pieris brassicae*

1.1 Economy, biology and ecology of *P. brassicae*

P. brassicae, is a butterfly native to Europe and Asia, often found in agricultural areas, meadows and parklands. The butterfly was first found in Nelson, New Zealand in May 2010, where it is classed as an unwanted pest due to the risk it poses to New Zealand native cress species [Phillips et al., 2014]. Following the discovery of this species, a concerted effort in between the Ministry of Primary Industry (MPI) and the Department of Conservation (DOC) has resulted in successfully preventing the spread of the butterfly in New Zealand. The species remain primarily of concern as it has the potential to cause huge economic losses in the form of brassica production (e.g. cabbage, Brussels sprouts, swede, turnip), as well as the destruction of endangered native cresses.

P. brassicae has two generations per year. It is naturally nomadic. Egg masses can contains around 40-100 eggs, which then explode outwards in strong migrations [Holland et al., 2006]. *P. brassicae* exhibits both local and long-distance dispersal. Feltwell [1982] concluded that the average local dispersal distances differed from a mere 7 m/year to a worrying 350 m/year. There is no evidence that *P. brassicae* exhibits directional bias during local dispersal [Davies and Gilbert, 1985]. Long-range movement is most often due to anthropogenic transportation, such as cars, cargo containers or host plants, of diapausing eggs and pupae providing a second mode of spread Feltwell [1982].

P. brassicae was chosen to investigate how different levels of complexity in the representation of realistic spatial patterns can change the final projected distribution of the species in Nelson area, New Zealand.

1.2 Study area

The extent of the study was delineated around Nelson port, New Zealand. The chosen spatial extent that roughly measures 35 × 40 km, covers a heterogeneous area both in terms of land cover and climatic composition.All datasets used for the simulation were clipped to the dimensions of this spatial extent. A common coordinate system of NZGD 2000 was used throughout the analyses. All external datasets were also re-projected to match the aforementioned coordinate system. The spatial resolution used for the simulations was 50 m. One time step represented on year.

1.3 Building a survival layer for *P. brassicae*

1.3.1 Growing degree days (GDD)

A raster layer that reflects the necessary base temperature to complete the *P. brassicae* life cycle within a season was sourced from Senay [2014]. The computed degree day data came in 100 m resolution and was re-sampled to 50 m resolution using the bilinear re-sampling technique. The GDD layer was extracted to the study area extent.

1.3.2 Land cover (LCC)

The New Zealand Land Cover Dataset, LCBD2 (Ministry for the Environment, 2004) was originally produced by Landcare Research based on SPOT imagery (resolution 15 m) and the pan-sharpened Landsat 7 ETM+ imagery (resolution 15 m). The dataset had 43 types of land covers. These were grouped and re-classed into six classes according to the reclassification keys given in Senay [2014]. The dataset was re-sampled to 50 m resolution using the bilinear re-sampling technique.

1.3.3 Local elevation variation (LEV)

Two granules of SRTM DEM data that cover our study area were downloaded from Reverb/ECHO NASA data portal. The datasets were then mosaicked and clipped to the study extent. The resulting continuous mosaicked dataset was projected from its source geographic coordinate system WGS 1984 into the NZGD 2000 projection to match the degree day and land use layers. The resolution of the SRTM dataset for the latitude where the study area is located is 80.5 m.

A focal 5×5 pixels elevation variation analysis was carried out to capture the local elevation gradient within each 1.6 km^2 area in the study extent.The resulting elevation difference layer revealed that there were 25 classes of gradient variations within the study area. The elevation variation layer was then re-sampled to 50m resolution using the bilinear re-sampling technique to match the GDD and LCC layers.

1.3.4 Landscape development

To investigate the effect of landscape composition and configuration on dispersal simulations, we designed two landscape types LS_1 and LS_2. The first landscape

included the GDD layer to enable us incorporate climatic survival requirement in our landscape and the LCC layer that allowed to differentiate land covers that are suitable to *P. brassicae.* The second landscape had the components of the first landscape, as well as the LEV layer that contributed to capturing the spatial pattern of the landscape in terms of local elevation variation at a higher definition than the other two components.

The landscape components were rescaled to produce a $0-1$ survival probability layer. Different criteria were used to rescale the individual components based on the range of values as well as the expert perceived effect of the components on the survival of *P. brassicae.* For the growing degree day dataset the layer was rescaled between $0.5-1$. The minimum probability of 0.5 was given to the GDD dataset as it requires 471.6 cumulative growing degree days for *P. brassicae* to complete its life cycle at least once. The range of GDD for the study area is $780-1295$, which means GDD will not be a limiting factor for *P. brassicae* survival within the considered landscape. The probability scheme used in Senay [2014] to assign survival percentages according to the land cover classes were applied to rescale the LCC layer.The values in the LEV layer were rescaled between the ranges $100-0$, where high local variation is given low survival probability (Table 1).

Both landscape LS_1 and LS_2 were characterized by two commonly used landscape metrics generated by the computer program FRAGSTATS 4.2 [McGarigal et al., 2012]. The percentage of suitable habitat cover (PLAND) was used to quantify the proportional abundance of each class type in the landscape (measure of habitat composition), the number of patch of each class type (NP), while the connectance index (CONNECT) was used to measure the connectivity between patch

type.These metrics were calculated as following:

$$PLAND = 100 * \frac{\sum_{j=1}^{n} a_{ij}}{A} \quad (1)$$

where a_{ij} is the area of patch ij and A is the total landscape area

$$NP = n_i \quad (2)$$

where n_i is the number of patches in the landscape of patch type (class) i.

$$CONNECT = 100 * \frac{\sum_{i=1}^{m} \sum_{j \neq k}^{n} c_{ijk}}{\sum_{i=1}^{m} (n_i(n_i - 1)/2)} \quad (3)$$

where c_{ijk} is equal to 1 if a functional joining between same patch type exists, based on a user-specific threshold distance (equal to 3 raster cells in this study) and 0 otherwise, and n_i is the number of patches in the landscape of patch type (class) i.

In this study, all metrics were calculated based on the distribution of raster cells within a specified distance of a focal point and an eight-neighbour rule. The connectance was calculated using the average Euclidean distance from cell centre to cell centre.More details about the metrics and their calculation can be found in [McGarigal et al., 2012]

1.4 Parametrizing the short and long-distance dispersal sub-model for *P. brassicae*

Following Senay [2014], the median distance of local movements of *P. brassicae* was approximated at 100m/year. A Von Neumann shape with range = 2 (100 m) was chosen to represent the uniform spread of *P. brassicae* within one time step. The initial dispersal site was arbitrarily set to a suitable patch around Nelson port, New Zealand.

The Cauchy distribution, commonly used to account for the fat-tailed characteristics of the distribution of rare long distance events [Kot et al., 1996, Cain et al., 2000, Higgins et al., 2000], was chosen to approximate the long-distance dispersal behaviour of *P. brassicae*. The Cauchy probability density function is given below.

$$f(x|x_0, \gamma) = \frac{1}{\pi}\left[\frac{\gamma}{(x - x_0)^2 + \gamma^2}\right] \tag{4}$$

where x_0 is the location parameter, specifying the location of the peak of the distribution and is used as a proxy of the median distance of long-distance dispersal events, and γ is the scale parameter of the Cauchy distribution. Each colony (occupied cell) was assumed to give rise to a Poisson number of offspring colonies that were initiated with a random fraction of the parent's abundance (stochastic long-distance jumps). Based on the study of Senay [2014], we fixed the median of long-dispersal distance at 24 752 m, the scale of the Cauchy distribution at 11 894 m and frequency of long dispersal at 0.41.

1.5 Parameter summary for *P. brassicae*

The parameters used to calibrate the dispersal in this study are given in Table 2. Twenty years of simulations were undertaken representing dispersal from the year 2010 to 2030, one model time step represents 6 months. The simulation was replicated 500 times to account for dispersal stochasticity) [Pitt, 2008].

2 Modelling dynamic range expansion of *Lymantria dispar*

2.1 Economy, biology and ecology of *L. dispar*

L. dispar, commonly know as the Asian Gypsy moth, is considered internationally to be among the most serious of all forest insect pests [Liebhold et al., 1992]. Their presence can destroy the aesthetic beauty of an area by causing large-scale defoliation and occasionally intense tree mortality, and covering the area with their waste products and silk. The species originally evolved in the temperate forests of Europe and Asia but was accidentally introduced to North America outside of Boston, Massachusetts, USA, in 1869. Since then it has subsequently invaded much of the susceptible forest of north-eastern North America, from Ontario to North Carolina and Nova Scotia to Wisconsin [Liebhold and Mastro, 1989, Morin et al., 2005]. The spread of the gypsy moth across eastern North America is, one of the most thoroughly studied biological invasion, providing a unique opportunity to explore spatio-temporal variability in rates of spread [Johnson et al., 2006].

The gypsy moth has one generation per year with four main life-stages. In spring the eggs hatch into larvae, which crawl up to the tree tops, then suspend themselves on silk threads and are passively dispersed by the wind [Liebhold et al., 1992]. Adult female gypsy moths are flightless, and ballooning of 1st instars usually occurs only over short distances. Long-range movement is most often due to anthropogenic transportation of life-stages, providing a second mode of spread [Liebhold and Tobin, 2010]. This leads to stratified diffusion. Whilst natural dispersal is limited to early instars, artificial dispersal affects all life-stages but most frequently

involves egg masses. Long distance dispersal leads to the formation of isolated colonies ahead of the initially infested area, which may grow and coalesce thereby increasing the rate of spread. By analysing available historical country level quarantine data on gypsy moth invasion, [Liebhold et al., 1992] concluded that the spread rates differed, throughout the past century, from a mere 2.82 km/year to a worrying 20.78 km/year. More recently, the value of 0.003 km^2/generation was referred to by several authors [e.g. Liebhold and Tobin, 2006, Robinet et al., 2008] sparking an inconsistency in terms of units. Finally, Tobin et al. [2007], using various spread rate estimation techniques, came up with even a broader range of spread rates as 2.6–28.6 km/year.

Another factor shown to affect the gypsy moth spread is the Allee effect [e.g. Liebhold and Bascompte, 2003, Vercken et al., 2011]. New isolated colonies of gypsy moth ahead of the initially infested area, are of low abundance and highly prone to Allee effects and extinction [Liebhold and Bascompte, 2003]. However, estimates of the Allee threshold are usually approximate: high observation error and demographic stochasticities are inevitable consequences of low abundance [Johnson et al., 2006, Vercken et al., 2011]. The current gypsy moth containment programme offers an exception because of its extensive grids of pheromone-baited traps, which are sensitive to extremely low moth densities along the invasion front. In Wisconsin, Allee effect threshold value was estimated at 2.2 moths/trap [Vercken et al., 2011]. A much higher value of 20.7 moths/trap was established in West Virginia and North Carolina. Johnson et al. [2006] provided overall estimates of 17 moths/trap for the Allee threshold.

The programme also allows to estimate habitat carrying capacity. Tobin et al.

[2007] pinpoint the carrying capacity at around 283 moths/trap in Wisconsin and 673 moths/trap in West Virginia and North Carolina. In earlier studies, Dwyer and Elkinton [1993] estimated the Allee threshold as 500 larvae/m^2 and Sharov and Liebhold [1998] as 200,000 egg masses/km^2. The latest values were difficult to compare with the estimates mentioned above as it refers to a different life-stage. Johnson et al. [2006] provided overall estimates of 687 moths/trap for the carrying capacity. In a similar study, having analysed pheromone trapping data from a large-scale field study in Washington, Liebhold and Bascompte [2003] estimated the Allee threshold as 106.7 moths/colony, which is much higher than the estimate by Johnson et al. [2006].

Prof. A. Liebhold suggested that the considerable variation reported for Allee thresholds, population dynamics and dispersal abilities can reflect geographical variation in the habitat that affects growth rates, dispersal rates and carrying capacity (pers. comm.). For analysing the spread of the Asian gypsy moth, Prof. A. Liebhold suggested to use the range of value given in Johnson et al. [2006]. These parameters were estimated using the data from the expanding gypsy moth population front - the so-called transition zone. Most of the other values were estimated from the state of Washington which is far from the gypsy moth invasion front, but where the insect is occasionally accidentally introduced and forms isolated colonies that must be eradicated.

2.2 Building a survival layer for *L. dispar*

We used the computer program Qrule 4.2 to generate binary (suitable, unsuitable) landscapes, in which fragmentation (measured as the degree of spatial autocorrela-

tion) and proportion of suitable habitat cover can be systematically and independently controlled [Gardner and Urban, 2007]. Qrule uses a midpoint displacement algorithm [Saupe, 1988] to generate multi-fractal maps in which the degree of spatial autocorrelation among adjacent cells (H) can be controlled. We generated landscape across a three-step gradient in spatial autocorrelation ($H = 0.3, 0.5, 0.7$) and a three-step gradient in the proportion of suitable habitat cover ($P = 35, 50, 75$), with 10 replicate landscapes for each factor combinations. The extent of the study covers $128x \times 128$ raster grid cells (13,384 sq.).

Each sample landscape was characterized by two commonly used landscape metrics generated by the computer program FRAGSTATS 4.2 [McGarigal et al., 2012]. The percentage of suitable habitat cover (PLAND) was used to quantify the proportional abundance of each patch type in the landscape (measure of habitat composition), while the connectance index (CONNECT) was used to measure the connectivity between suitable patches. In this study, all metrics were calculated based on the distribution of raster cells within a specified distance of a focal point and an eight-neighbour rule. The connectance was calculated using the average Euclidean distance from cell centre to cell centre.

2.3 Parametrizing the local dispersal sub-model for *L. dispar*

A Von Neumann shape with range = 1 was chosen to represent the uniform, local neighbourhood for spread of *L. dispar* within one time step. We chose a cell resolution of 10 km/year to approximate the median distance of local movements of larvae and adults as shown in in Johnson et al. [2006] and [Liebhold et al., 1992].

2.4 Parametrizing the long-distance dispersal sub-model for *L. dispar*

Long-distance dispersal events were approximated by a Cauchy probability distribution. Each colony (occupied cell) was assumed to give rise to a Poisson number of offspring colonies that were initiated with a random fraction of the parent's abundance (stochastic long-distance jumps). Based on the study of Johnson et al. [2006], we fixed the median of long-dispersal distance at 50 km and frequency of long dispersal at 0.01.

2.5 Parametrizing the population sub-model for *L. dispar*

Following Johnson et al. [2006] and Liebhold and Bascompte [2003], the abundance of gypsy moth was approximated by a deterministic Allee logistic growth model:

$$N_{t+1} = N_t \exp\left[r\left(1 - \frac{N}{K}\right)\left(\frac{N_{t-1} - C}{K}\right)\right] \quad (5)$$

where N is the number of individuals at time t, C is the Allee threshold, r the intrinsic growth rate and K the carrying capacity. The values of these parameters were based on previous estimates reported in Johnson et al. [2006] and Liebhold and Bascompte [2003] to fit release-capture data collected from 1988 to 2004.

2.6 Parameter summary for *L. dispar*

The MDiG parameters used in this study are given in Table 3. The analysis was approached from a case-specific view point, parametrising a population sub-model based on detailed demographic and dispersal attributes of the well studied Euro-

pean gypsy moth, *L. dispar.* In this way, our model had sufficient biological details to reproduce realistic parameter ranges. MDiG was then used to explore four different scenarios of demographic and dispersal behaviours in changing environments (Table 3): a specie with 1) slow reproducing and short dispersal abilities, 2) slow reproducing and long dispersal abilities, 3) fast reproducing and short dispersal abilities and, 4) fast reproducing and short dispersal abilities. The main reason for doing this was to evaluate how changes in the intrinsic growth rate, median distance of long-distance dispersal events and landscape structure affect the population density – d (number of individuals per raster cells) and the rate of spread – ROS (number of new cells occupied per simulation run). Hundred years of simulations were undertaken representing dispersal, one model time step represents one year. Simulations were replicated 500 times to account for dispersal stochasticity.

References

Michael L Cain, Brook G Milligan, and Allan E Strand. Long-distance seed dispersal in plant populations. *American Journal of Botany*, 87(9):1217–1227, 2000.

CR Davies and N Gilbert. A comparative study of the egg-laying behaviour and larval development of *Pieris rapae L.* and *P. brassicae L.* on the same host plants. *Oecologia*, 67(2):278–281, 1985.

Greg Dwyer and Joseph S Elkinton. Using simple models to predict virus epizootics in gypsy moth populations. *Journal of Animal Ecology*, 62(1):1–11, 1993.

John Feltwell. *Large White butterfly: the biology, biochemistry, and physiology of*

Pieris brassicae (Linnaeus), volume 18. Springer Science and Business Media, 1982. 564 pp.

Robert H. Gardner and Dean L. Urban. Neutral models for testing landscape hypotheses. *Landscape Ecology*, 22(1):15–29, 2007.

Steven I Higgins, David M Richardson, and Richard M Cowling. Using a dynamic landscape model for planning the management of alien plant invasions. *Ecological Applications*, 10(6):1833–1848, 2000.

Richard A Holland, Martin Wikelski, and David S Wilcove. How and why do insects migrate? *Science*, 313(5788):794–796, 2006.

Derek M. Johnson, Andrew M. Liebhold, Patrick C. Tobin, and Ottar N. Bjørnstad. Allee effects and pulsed invasion by the gypsy moth. *Nature*, 444(7117):361–363, 2006.

Mark Kot, Mark A Lewis, and Pauline van den Driessche. Dispersal data and the spread of invading organisms. *Ecology*, 77(7):2027–2042, 1996.

Andrew Liebhold and Jordi Bascompte. The Allee effect, stochastic dynamics and the eradication of alien species. *Ecology Letters*, 6(2):133–140, 2003.

Andrew Liebhold and Victor Mastro. Learning from the legacy of Leopold Trouvelot. *Bulletin of the Entomological Society of America*, 35(2):20–22, 1989.

Andrew M. Liebhold and Patrick C. Tobin. Growth of newly established alien populations: comparison of North American gypsy moth colonies with invasion theory. *Population Ecology*, 48(4):253–262, 2006.

Andrew M. Liebhold and Patrick C. Tobin. Exploiting the Achilles heels of pest invasions: Allee effects, stratified dispersal and management of forest insect establishment and spread. In *New Zealand Journal of Forestry Science*, volume 40, pages 25–33. New Zealand Forest Research Institute, 2010.

Andrew M. Liebhold, Joel A. Halverson, and Gregory A. Elmes. Gypsy moth invasion in North America: a quantitative analysis. *Journal of Biogeography*, 19(5): 513–520, 1992.

K McGarigal, S Cushman, and E Ene. FRAGSTATS v4: spatial pattern analysis program for categorical and continuous maps., 2012. URL `http://www.umass.edu/landeco/research/fragstats/fragstats.html`.

Randall S. Morin, Andrew M. Liebhold, Eugene R. Luzader, Andrew J. Lister, Kurt W. Gottschalk, and Daniel B. Twardus. Mapping host-species abundance of three major exotic forest pests. Technical Report NE-726, Department of Agriculture, Forest Service, Northeastern Research Station, Newtown Square, PA: U.S., 2005.

CB Phillips, K Brown, C Green, G Walker, K Broome, R Toft, B Vander Lee, and M King. *Pieris brassicae* (great white butterfly) eradication annual report. Technical report, Whakatū/Nelson Office, Department of Conservation, New Zealand (Report for Ministry for Primary Industries), 2014. 37 pp.

Joel Peter William Pitt. *Modelling the spread of invasive species across heterogeneous landscapes.* PhD, Lincoln University, New Zealand, 2008. 232 pp.

Christelle Robinet, D. R. Lance, K. W. Thorpe, K. S. Onufrieva, P. C. Tobin, and A. M. Liebhold. Dispersion in time and space affect mating success and Allee

effects in invading gypsy moth populations. *Journal of Animal Ecology*, 77(5): 966–973, 2008.

Dietmar Saupe. Algorithms for random fractals. In *The science of fractal images*, pages 71–136. Springer, 1988.

Senait Dereje Senay. *Modelling invasive species-landscape interactions using high resolution, spatially explicit models*. PhD, Lincoln University, New Zealand, 2014. 352 pp.

Alexei A Sharov and Andrew M Liebhold. Model of slowing the spread of gypsy moth (*Lepidoptera: Lymantriidae*) with a barrier zone. *Ecological Applications*, 8 (4):1170–1179, 1998.

Patrick C. Tobin, Andrew M. Liebhold, and E. Anderson Roberts. Comparison of methods for estimating the spread of a non-indigenous species. *Journal of Biogeography*, 34(2):305–312, 2007.

E. Vercken, A. M. Kramer, P. C. Tobin, and J. M. Drake. Critical patch size generated by Allee effect in gypsy moth, *Lymantria dispar (L.)*. *Ecology Letters*, 14(2):179–186, 2011.

Attributes	Growing degree days (GDD)	Land cover classes (LCC)	Local Elevation Variation (LEV)
Value	Growing degree day	Nominal land cover classes	Ordinal difference in elevation from surrounding cells
Original resolution	20 km	15 m	3 arc seconds
Range	$780 - 1295$	$1 - 6$	$1 - 25$
Rule	Rescaled between $50 - 100$	$1 = 90; 2 = 80; 3 = 50; 4 = 30; 5 = 10; 6 = 0$ following Senay [2014]	Rescaled between $100 - 0$; where high variations were given low survival percentage
Included in	LS_1 & LS_2	LS_1 & LS_2	LS_2

Table 1: Survival layer construction for *P. brassicae*

**Parameters**	**Abbreviated code**	**Parameters value**
**Population sub-model**		
Presence/absence model		
**Local dispersal sub-model** (uniform)		
Mean distance of local dispersal events	R	100 meters
**Long-distance dispersal sub-model** (Cauchy distribution)		
Median distance of long dispersal events	x_0	24 752 meters
Cauchy scale	λ	11 894
Frequency of long dispersal events	f	0.41
**Propagule pressure**		
Propagule size	nbp	1 presence cell
**Landscape structure**		
Survival layer excluding gradient level variations	LS_1	
Survival layer including gradient level variations	LS_2	

Table 2: Baseline value of MDiG parameters for modelling the spread of the great white butterfly *P. brassicae*, in computer realistic landscapes.

Parameters	Abbreviated code	Parameters value
**Population sub-model** (logistic model wit Allee effect - Equation 5)		
Growth rate	r	0.815 (slow) per capacity growth
		1.223 (fast) per capacity growth
Allee threshold	C	2 individuals per raster cell
Carrying capacity	K	50 individuals per raster cell
**Local dispersal sub-model** (uniform)		
Mean distance of local dispersal events	R	1 raster cell (10 km)
**Long-distance dispersal sub-model** (Cauchy distribution)		
Median distance of long dispersal events	λ	3 (short) raster cells
		5 (long) raster cells
Frequency of long dispersal events	f	0.05
**Propagule pressure**		
Propagule size	nbp	5 individuals
**Landscape structure** (binary landscapes)		
Spatial autocorrelation (fragmentation)	H	0, 0.5, 1
Percentage of suitable habitat cover	P	25, 50, 75

Table 3: Baseline value of MDiG parameters for modelling the spread of gypsy moth, *L.dispar*, in computer generated landscapes.
